# Sepsis-Specific Risk Factors for Augmented Renal Clearance (ARC) and the Effect of Inflammation on Duration of ARC in Patients with Sepsis: A Single-Center Retrospective Cohort Study

**DOI:** 10.3390/jcm15145662

**Published:** 2026-07-19

**Authors:** Shogo Adachi, Masayuki Ohbayashi, Gen Inoue, Keisuke Suzuki, Miki Sato, Kenji Dohi, Mari Kogo

**Affiliations:** 1 Division of Pharmacotherapeutics, Department of Clinical Pharmacy, Showa Medical University School of Pharmacy, 1-5-8 Hatanodai, Shinagawa-ku, Tokyo 142-8555, Japan; ohbayashi@pharm.showa-u.ac.jp (M.O.);; 2 Department of Emergency and Critical Care Medicine, Showa Medical University Hospital, 1-5-8 Hatanodai, Shinagawa-ku, Tokyo 142-8666, Japan; gen.inoue@med.showa-u.ac.jp (G.I.); kdop@med.showa-u.ac.jp (K.D.); 3 Department of Emergency and Disaster Medicine, Showa Medical University School of Medicine, 1-5-8 Hatanodai, Shinagawa-ku, Tokyo 142-8555, Japan

**Keywords:** augmented renal clearance, sepsis, antimicrobial stewardship, risk factor, critical care

## Abstract

**Background/Objectives:** Augmented renal clearance (ARC) may reduce blood concentrations of renally eliminated antibiotics and increase the risk of treatment failure in sepsis. We investigated the risk factors for ARC and the effect of inflammation on ARC duration. **Methods:** This retrospective cohort study included 282 patients treated for sepsis. The primary endpoint was the ARC incidence, while the secondary endpoints were the relationship between the ARC duration and the time courses of inflammatory marker levels. Patient characteristics, disease severity, laboratory data, and treatment data were collected from medical records. Risk factors for ARC were identified using logistic regression. The effect of the inflammatory response on the ARC duration was evaluated using the Mann–Whitney U test. **Results:** Among 174 patients (median age, 78 years [range: 29–95]; 94 [54.0%] male; the median intensive care unit length of stay was 6 days [1–76 days]) with ARC identified in 15.5% of patients. Significant risk factors for ARC were age < 75 years (odds ratio, 4.748), albumin levels < 3.0 g/dL (3.672), serum potassium levels < 3.6 mmol/L (5.196), and sequential organ failure assessment scores ≤ 9 (3.803). Patients with an ARC duration of ≥ 8 days had significantly higher white blood cell counts and C-reactive protein levels at ARC incidence than those with an ARC duration < 8 days. **Conclusions:** Age, albumin, serum potassium, and sequential organ failure assessment scores were risk factors for ARC in patients with sepsis. Persistent inflammation may prolong ARC. These findings can aid in choosing antibiotic dosing regimens based on renal function in patients with sepsis at high risk of ARC.

## 1. Introduction

Sepsis is a life-threatening organ dysfunction caused by a dysregulated host response to infection [1]. Sepsis affects millions of people worldwide annually [2,3,4], with a mortality rate of 15.0–26.7%. Appropriate antimicrobial administration is crucial for reducing mortality in patients with sepsis [1].

Beta-lactam antibiotics, commonly used to treat sepsis, exhibit time-dependent antibacterial activity that is enhanced by maximizing the time during which their blood concentration remains above the minimum inhibitory concentration (T > MIC) [5]. Beta-lactam antibiotics are predominantly excreted via the kidneys; thus, T > MIC is strongly influenced by renal function [6,7].

Some patients with sepsis may develop augmented renal clearance (ARC), which is defined as a glomerular filtration rate (GFR) > 130 mL/min/1.73 m^2^ [2,8,9]. Sepsis-induced severe inflammation causes increased cardiac output and reduced vascular resistance. Consequently, renal blood flow increases, which may induce ARC [6,10].

In patients with ARC, increased excretion of renally eliminated antibiotics reduces antibiotic blood concentrations, potentially increasing the risk of treatment failure [6,10]. Therefore, appropriate antibiotic dosing regimens for patients with ARC—such as dose escalation, prolonged infusion, and continuous infusion—have been extensively investigated [11,12,13]. To administer appropriate antibiotic dosing regimens for patients with ARC, it is necessary to clarify the risk factors for ARC and the time course of renal function.

In various patient populations—including those with postoperative status, respiratory failure, central nervous system disease, or cardiovascular disease—several risk factors for ARC have been reported, such as younger age, male sex, trauma, mechanically assisted ventilation, and vasopressor use [14,15]. However, risk factors for ARC specific to patients with sepsis remain unclear. Sepsis is characterized by organ dysfunction, severe inflammation, and altered hemodynamics. Therefore, previously reported risk factors may not adequately reflect the unique pathophysiology and clinical characteristics of patients with sepsis.

In one study, among critically ill patients admitted to the intensive care unit (ICU), the median duration of ARC was 5 days [14]. In patients with sepsis, severe inflammation may cause fluctuations in renal function, potentially inducing ARC and prolonging its duration [6,16]. Prolonged ARC can shorten the T > MIC of the antibiotic, thereby compromising its clinical efficacy. However, the relationship between the intensity of inflammation and the duration of ARC remains unclear. Clarifying the risk factors for ARC in patients with sepsis and the effect of inflammation on the duration of ARC may facilitate earlier optimization of antibiotic dosing regimens. This may ultimately lead to a reduced hospital stay and decreased mortality in septic patients with ARC. Therefore, to help guide appropriate antibiotic dosing regimens, we investigated risk factors for ARC and the effect of inflammation on ARC duration in patients with sepsis.

## 2. Materials and Methods

### 2.1. Study Design and Patients

**Figure 1:**
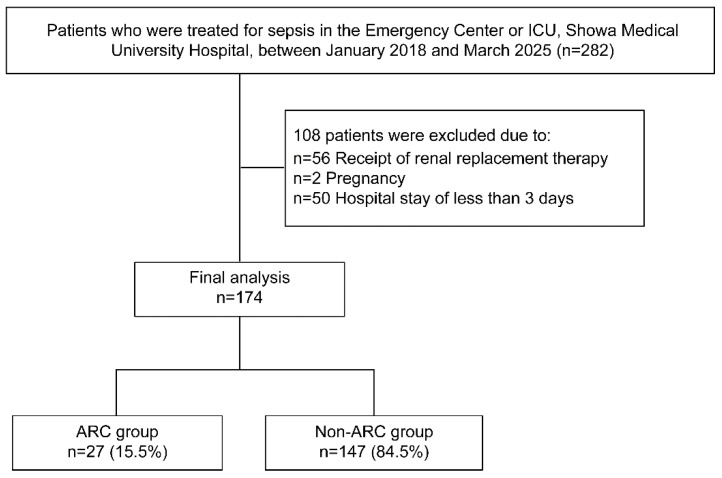
Flowchart of eligible patients. ICU, intensive care unit; ARC, augmented renal clearance.

In this retrospective cohort study, we included 282 patients who were treated for sepsis in the Emergency Center or ICU at Showa Medical University Hospital between January 2018 and March 2025. The inclusion criteria were: (1) age ≥ 18 years and (2) diagnosis of sepsis based on the Sepsis-3 definition [17] on the day of admission. The exclusion criteria were: (1) receipt of renal replacement therapy (*n* = 56), (2) pregnancy (*n* = 2), and (3) hospital stay < 3 days (*n* = 50). After excluding 108 patients, 174 patients were included in the final analysis (Figure 1).

### 2.2. Sepsis Management

Sepsis was managed following clinical practice guidelines [1,18,19]. The initial treatment interventions included empiric antimicrobial therapy, intravenous fluid resuscitation, and vasopressor support. Pharmacological and non-pharmacological interventions during hospitalization were performed at the discretion of the attending physicians, based on the patient’s clinical condition.

### 2.3. Data Collection

Data on patient characteristics, laboratory parameters, disease severity, treatments, and clinical outcomes were collected from patient medical records. Patient characteristics included age, sex, body mass index (BMI), medical history, and infection site at admission. Laboratory data collected at admission included white blood cell (WBC) count, red blood cell count, platelet count, estimated GFR (eGFR), pH, and hemoglobin, hematocrit, total protein, albumin (Alb), total bilirubin, serum sodium, serum potassium, serum creatinine (SCr), blood urea nitrogen (BUN), C-reactive protein (CRP), and lactate levels. The eGFR, WBC count, and SCr and CRP levels were monitored for 14 days after admission. The severity of sepsis was assessed using the sequential organ failure assessment (SOFA) score [20] at admission.

Treatments included the use of vasopressors and mechanical ventilation. Clinical outcomes included the duration of vasopressor use, the duration of mechanical ventilation, ICU length of stay, and in-hospital mortality.

### 2.4. Endpoints

The primary endpoint was the incidence of ARC within 14 days of hospitalization. ARC was defined as an eGFR > 130 mL/min/1.73 m^2^ [9]. SCr levels were measured using an enzymatic method. The eGFR was calculated using a three-variable estimation equation developed in Japanese individuals [21]:

eGFR (mL/min/1.73 m^2^) = 194 × SCr^−1.094^ × Age^−0.287^ (×0.739 if female)

The secondary endpoints were the duration of ARC and the time courses of inflammatory marker levels. Persistent ARC was defined as the presence of ARC on the day after onset. In contrast, non-persistent ARC was defined as the absence of ARC for 2 consecutive days after the day of onset. The duration of ARC was calculated as the total number of days on which ARC was observed during the 14-day observation period.

### 2.5. Statistical Analysis

#### 2.5.1. Sample Size

We estimated that a sample size of 77 patients would be required to detect an odds ratio of 2.0, with 80% statistical power at a 0.05 significance level, under the assumption of an ARC incidence of 33.0% [8]. When recalculated using the actual ARC incidence of 15.5%, the required sample size was 128 patients. Therefore, the sample size in this study was considered sufficient for the analyses [22].

#### 2.5.2. Stratification

Continuous variables were dichotomized into categorical variables, consistent with previous studies on ARC, for practical clinical application [23,24]. Categories were divided based on cutoff values for age (75 years) and hemoglobin (13 and 12 g/dL in males and females, respectively), using values proposed by the World Health Organization. Further variables were dichotomized as follows: Alb levels (cutoff of 3.0 g/dL), following the reference value for the management target for patients with sepsis [1]; pH (cutoff: 7.35), following the reference value for acidosis [25]; CRP (cutoff: 15.16 mg/dL); and SOFA score (cutoff: 9), using the Youden index method [26]. The other laboratory variables were dichotomized based on the upper or lower boundaries of the reference range values for clinical items published by the Japanese Clinical Laboratory Standards Council [27].

#### 2.5.3. Time Course of Renal Function Data

The time course of eGFR in the ARC and non-ARC groups was compared using the Mann–Whitney U test. Because this was an exploratory study, no adjustments were made for multiple comparisons.

#### 2.5.4. Risk Factors for ARC

Categorical variables are presented as numbers and percentages, and continuous variables as medians (min–max). Patient characteristics, clinical findings, laboratory data, and disease severity were compared between the ARC and non-ARC groups. The chi-square or Fisher’s exact test was used for univariate analysis, and significant variables were entered into a multivariate analysis. Although age is a component of the eGFR equation, it was included as a covariate in the multivariable logistic regression analysis because previous studies have identified it as a risk factor for ARC [8]. SCr was excluded, as it is a component of the SOFA score. Although BUN levels and SOFA scores were not strongly correlated (r = 0.238), SOFA scores were selected as a standard tool for diagnosing and assessing sepsis severity [1]. Finally, the following variables were entered into the multivariate analysis: age < 75 years, Alb < 3.0 g/dL, serum potassium < 3.6 mmol/L, and SOFA score ≤ 9.

As an exploratory analysis, a multivariate logistic regression analysis using a stepwise method was performed to identify independent risk factors for the incidence of ARC. Because the median time from admission to ARC incidence was 5 days in this study, we considered the influence of time to be limited and therefore used logistic regression analysis. The variance inflation factor was calculated for each variable included in the multivariable analysis, and all variance inflation factor values were below 2, indicating no evidence of multicollinearity among the variables. The predictive accuracy for the incidence of ARC was evaluated using receiver operating characteristic curve analysis. Missing values were not imputed. Statistical significance was set at *p* < 0.05. Statistical analyses were performed using SPSS version 28 (IBM Corp., Armonk, NY, USA).

#### 2.5.5. Comparison of Treatments and Clinical Outcomes Between the ARC and Non-ARC Groups

As an exploratory analysis, treatments and in-hospital mortality were compared between the ARC and non-ARC groups using the chi-square test. The duration of vasopressor use, duration of mechanical ventilation, and ICU length of stay were also compared between the ARC and non-ARC groups using the Mann–Whitney U test.

#### 2.5.6. Duration of ARC

To estimate the median duration of ARC, a Kaplan–Meier curve was constructed. In this analysis, the day of ARC incidence was defined as day 0, and patients were assessed consecutively during the observation period. Resolution of ARC was defined as non-ARC status on two consecutive days. Patients were censored at the time of death, discharge, or the end of the observation period. Missing values were not imputed.

#### 2.5.7. Effect of Inflammation on ARC Duration

Patients were divided into two groups according to the calculated median ARC duration (8 days). WBC counts and CRP levels were compared between patients with an ARC duration of ≥8 days and <8 days using the Mann–Whitney U test. Because this was an exploratory study, no adjustments were made for multiple comparisons.

## 3. Results

### 3.1. Patient Characteristics

Patient characteristics are presented in Table 1. The median age was 78 years (range: 29–95), and 94 (54.0%) were male. The median SOFA score at admission was 8 (1–18). The median SCr level and eGFR at admission were 1.41 (0.26–8.10) mg/dL and 34.1 (4.1–173.1) mL/min/1.73 m^2^, respectively. The median WBC count and CRP level at admission were 11.9 (0.2–50.6) × 10^3^/µL and 12.81 (0.04–55.73) mg/dL, respectively. The urinary tract was the most common site of infection (35.1%). Regarding clinical outcomes, the median ICU length of stay was 6 days (1–76), and in-hospital mortality was 14.9%.

### 3.2. Endpoints

ARC occurred in 27 patients (15.5%; Figure 1). The median time to ARC incidence was 5 days (2–13). Among patients with ARC, the median eGFR on the day of ARC incidence was 142.3 (131.5–218.7) mL/min/1.73 m^2^.

### 3.3. Time Course of Renal Function

**Figure 2:**
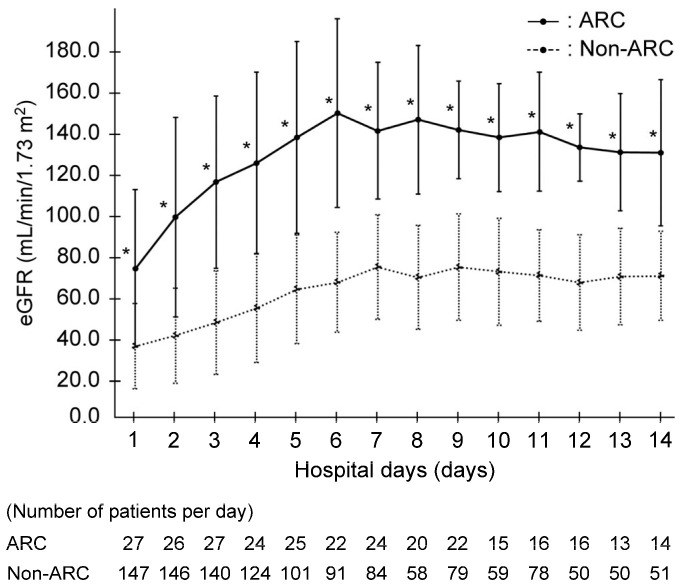
Time course of renal function in patients with sepsis. The admission day was defined as day 1. The eGFR over the 14-day observation period is plotted for the ARC and non-ARC groups. Data are presented as the mean ± standard deviation. Error bars indicate the standard deviation. The eGFR values remained significantly higher in the ARC group than in the non-ARC group from the day of admission through day 14. ARC, augmented renal clearance; eGFR, estimated glomerular filtration rate; * *p* < 0.05.

Figure 2 shows the time course of renal function in the ARC and non-ARC groups. The eGFR values remained significantly higher in the ARC group than in the non-ARC group from the day of admission through day 14.

### 3.4. Comparison of Patient Characteristics Between the ARC and Non-ARC Groups

Table 2 shows the comparison of patient characteristics between the ARC and non-ARC groups. The proportions of patients with BMI ≥ 18.5 kg/m^2^ and BUN > 21 mg/dL were significantly lower in the ARC group. Similarly, the ARC group had a significantly lower proportion of patients with elevated SCr (males > 1.063 mg/dL, females > 0.79 mg/dL). Conversely, the proportions of patients with age < 75 years, Alb < 3.0 g/dL, serum potassium < 3.6 mmol/L, and SOFA score ≤ 9 were significantly higher in the ARC group than in the non-ARC group.

### 3.5. Risk Factors for the Incidence of ARC

Age < 75 years, Alb < 3.0 g/dL, serum potassium < 3.6 mmol/L, and SOFA score ≤ 9 were independently associated with the incidence of ARC (Table 3). The result of the receiver operating characteristic curve analyses is shown in Supplementary Table S1.

### 3.6. Comparison of Treatments and Clinical Outcomes Between the ARC and Non-ARC Groups

The ICU length of stay was significantly longer in the ARC group than in the non-ARC group. No significant difference in in-hospital mortality was observed between the two groups (Supplementary Table S2).

### 3.7. Duration of ARC

**Figure 3:**
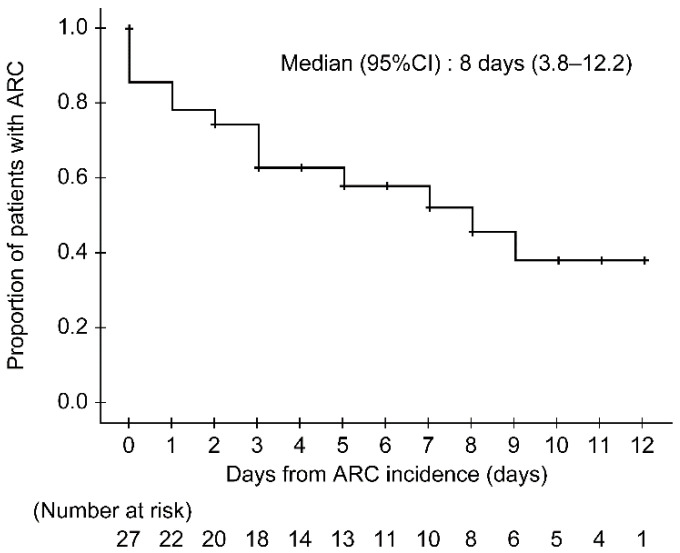
Cumulative persistence rate of ARC in patients with sepsis (*n* = 27). The day of the ARC incidence was defined as day 0. The proportion of patients with ARC over time is shown. ARC, augmented renal clearance; CI, confidence interval.

The Kaplan–Meier curve for ARC duration is shown in Figure 3. The median ARC duration was 8 days (95% confidence interval; 3.8–12.2).

### 3.8. Effect of Inflammation on the ARC Duration

**Figure 4:**
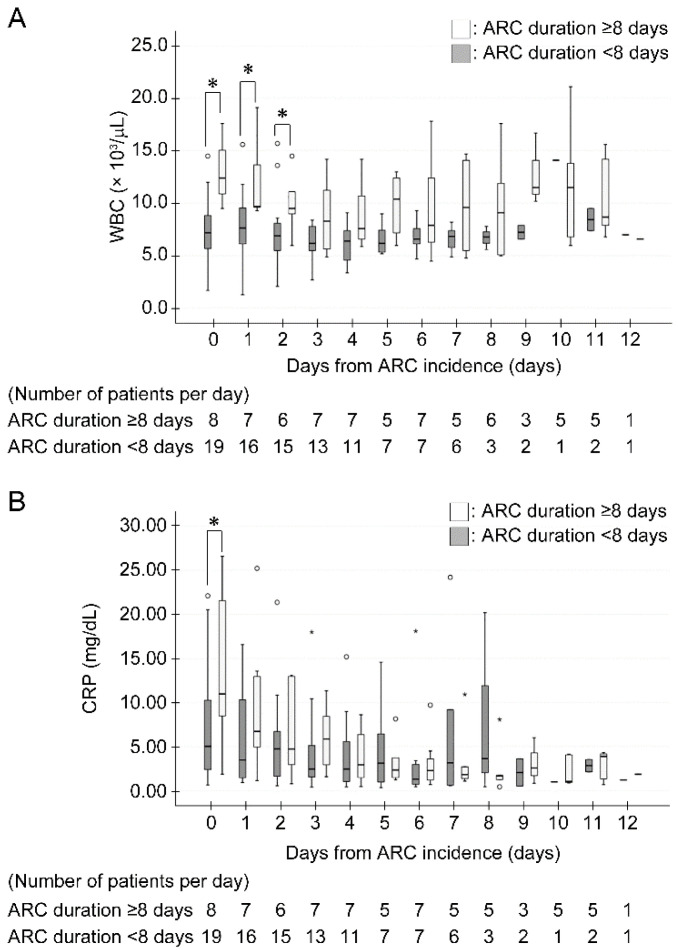
Effect of inflammation on the ARC duration in patients with sepsis (*n* = 27). (**A**) Relationship between WBC counts and ARC duration. (**B**) Relationship between CRP levels and ARC duration. The center line represents the median, the box represents the interquartile range, and the whiskers extend to the minimum and maximum values. ARC, augmented renal clearance; WBC, white blood cell; CRP, C-reactive protein; * *p* < 0.05.

The effect of inflammation on the duration of ARC is shown in Figure 4. WBC counts on days 0, 1, and 2 were significantly higher in the ARC duration ≥ 8 days group than in the <8 days group. Similarly, CRP levels on day 0 were significantly higher in the ARC duration ≥ 8 days group.

## 4. Discussion

Our findings revealed that age < 75 years, Alb < 3.0 g/dL, serum potassium < 3.6 mmol/L, and SOFA score ≤ 9 were independent risk factors for ARC in patients with sepsis. Additionally, the ARC duration was prolonged in patients with elevated WBC counts and CRP levels at ARC incidence. To the best of our knowledge, this is the first study to identify sepsis-specific risk factors for ARC and to evaluate the association between inflammation and ARC duration. These indicators may be useful for predicting the incidence and persistence of ARC in patients with sepsis. These indicators may also facilitate the selection of optimized antibiotic dosing regimens, thereby maximizing the T > MIC.

Previous studies on ARC have primarily targeted patients admitted to the ICU with various conditions, including sepsis, trauma, cardiovascular disease, and neurologic disease [14,15]. Sepsis is characterized by pronounced systemic inflammation, severe hemodynamic instability, and high mortality; accordingly, the ARC risk factors reported in previous studies may not adequately reflect sepsis-specific pathophysiological characteristics. We focused on patients with more severe sepsis than those in previous reports. Consequently, the risk factors for ARC identified in this study may reflect the severity of sepsis-associated organ dysfunction and systemic inflammation.

Prompt and appropriate antibiotic administration is important for improving clinical outcomes and reducing mortality in patients with sepsis. Previous studies have reported lower antibiotic concentrations in patients with sepsis and ARC [12,13]. ARC has also been associated with worse clinical outcomes [28]. Consistent with these observations, we found that ARC was associated with a significantly prolonged ICU length of stay. These findings have clinically significant implications, as they provide a rationale for reconsidering antibiotic dosing strategies and developing novel therapeutic approaches for patients with sepsis. It should be noted, however, that treatment failure in the ARC group may be influenced not only by the pathophysiological effect of ARC itself but also by patient characteristics and clinical status.

### 4.1. Incidence of ARC

A previous study involving a heterogeneous population of critically ill patients reported an ARC incidence of 24.9% [29]. In contrast, the incidence of ARC was 15.5% in the present study, which exclusively included patients with sepsis. This lower incidence may partly reflect the greater severity of organ dysfunction in this sepsis-specific population.

### 4.2. Risk Factors for ARC

Age < 75 years was independently associated with the incidence of ARC. Younger age has been previously identified as a risk factor for ARC [8,14,15], and it is a key variable in an ARC prediction score model developed in a Japanese ICU population [24]. Physiologically, younger patients possess renal functional reserve (RFR); thus, they are more likely to exhibit an increased GFR in response to elevations in cardiac output and renal blood flow induced by inflammation in sepsis [30,31,32]. Therefore, the greater preserved RFR in patients aged < 75 years may have contributed to the incidence of ARC.

Serum potassium < 3.6 mmol/L was an independent risk factor for the incidence of ARC. Elevated serum potassium levels are caused by several factors, including acid–base imbalance and reduced renal excretion. Although lactic acidosis is common in patients with sepsis, a decrease in pH due to lactic acidosis is unlikely to cause an increase in serum potassium levels [33,34]. Furthermore, the effect of respiratory acidosis is relatively modest [34]; thus, the contribution of pH changes to increased serum potassium levels is likely minimal. In this study, patients with serum potassium ≥ 3.6 mmol/L had significantly higher SCr and lower renal function than those with <3.6 mmol/L. Therefore, a serum potassium level ≥ 3.6 mmol/L may reflect reduced renal function, which may have contributed to the lower ARC incidence.

Hypoalbuminemia, associated with systemic inflammation in patients with sepsis, results from decreased protein synthesis and increased capillary permeability [35]. Reduced serum Alb levels decrease plasma colloid oncotic pressure, leading to a decrease in circulating plasma volume [36]. As a compensatory mechanism, sympathetic activation occurs, resulting in increased cardiac output and renal blood flow [37]. Therefore, the hemodynamic alterations secondary to hypoalbuminemia may have contributed to the incidence of ARC.

A previous study showed that a SOFA score ≤ 4 is a risk factor for ARC [23]. In addition, the incidence of ARC has been reported to decrease with increasing SOFA score [38,39]. Consistent with these findings, a positive correlation between the SOFA score ≤ 9 and ARC incidence was observed in this study. This is likely because patients with lower SOFA scores exhibit preserved renal blood flow resulting from less severe multiple organ dysfunction and circulatory failure. Therefore, the incidence of ARC may be higher in patients with SOFA scores ≤ 9.

Receiver operating characteristic curve analyses were performed for the combined multivariable model including all four variables. Notably, the area under the curve of 0.808 (Supplementary Table S1) obtained by combining these four identified risk factors was close to the area under the curve of 0.841 reported for a previously developed machine learning model [40]. However, direct comparisons should be made with caution due to methodological differences.

### 4.3. Comparison with Prior Reports on ARC Duration

The median duration of ARC in this study was 8 days, which is longer than the previously reported 5 days [13]. The patients in this study had higher SOFA scores, reflecting a more severely ill population, which may have influenced the duration of ARC. The incidence of ARC has been reported to be lower in patients with more severe sepsis than in those with less severe sepsis [36,37]. In contrast, ARC may persist in patients with preserved RFR because of inflammation-induced increases in renal blood flow. Taken together, systemic inflammation may play a complex role in both the incidence and persistence of ARC. Moreover, patients with severe sepsis often receive more aggressive fluid resuscitation and vasopressor therapy to stabilize hemodynamics; such intensive management may have contributed to the prolonged duration of ARC observed in this study.

### 4.4. Effect of Inflammation on ARC Duration

In the group with a longer ARC duration, the WBC count and CRP level were higher at ARC incidence, and these inflammatory markers remained elevated throughout the observation period. The release of inflammatory cytokines may reduce systemic vascular resistance and increase cardiac output, thereby enhancing renal blood flow and glomerular filtration [6,9]. The inflammation-driven hemodynamic changes may contribute to the persistence of ARC. Our findings suggest that the intensity and persistence of inflammation may be associated with a prolonged duration of ARC.

### 4.5. Limitations

This study had some limitations. First, the external validity of the identified risk factors could not be verified, as this was a single-center retrospective study. Second, renal function was estimated using eGFR; however, a more accurate assessment using measured creatinine clearance or inulin clearance was not feasible in this retrospective setting. Consequently, the ARC status of some patients may have been misclassified, potentially affecting the estimated incidence of ARC and the identification of risk factors. Third, baseline renal function data prior to admission were unavailable for most patients. Fourth, since inflammatory mediators such as IL-6 were not measured, the mechanistic impact of inflammation on renal function could not be evaluated. Finally, the impact of confounding factors—including the dosages and duration of medications, fluid balance, and other non-pharmacological interventions—was not fully accounted for in this study.

## 5. Conclusions

Age < 75 years, Alb < 3.0 g/dL, serum potassium < 3.6 mmol/L, and SOFA score ≤ 9 were identified as independent risk factors for ARC in patients with sepsis. These factors may be useful indicators for the early identification of patients with sepsis at high risk of ARC. Additionally, this study showed that persistent inflammation may prolong the ARC duration in patients with ARC. These findings may help guide antibiotic dosing based on renal function in patients with sepsis at high risk of ARC.

## Figures and Tables

**Table 1:** Patient characteristics (*n* = 174).

Variables	*N*	*n* (%) or Median (Range)	
Patient characteristics			
Male	174	94	(54.0)
Age (years)	174	78	(29–95)
BMI (kg/m^2^)	174	20.7	(11.9–49.2)
Comorbidities			
Hypertension	172	77	(44.8)
Dyslipidemia	172	51	(29.7)
Diabetes	172	33	(19.2)
Laboratory data			
WBC (×10^3^/µL)	174	11.9	(0.2–50.6)
RBC (×10^4^/µL)	174	385	(143–607)
Hb (g/dL)	174	12.0	(4.1–17.9)
Hematocrit (%)	174	36.2	(14.7–54.1)
Plt (×10^4^/µL)	174	16.9	(0.4–53.4)
Total protein (g/dL)	174	6.1	(3.7–8.5)
Alb (g/dL)	173	2.9	(1.0–4.7)
T-Bil (mg/dL)	174	1.0	(0.2–13.1)
Sodium (mmol/L)	174	137.3	(106.4–189.0)
Potassium (mmol/L)	174	4.0	(2.2–7.4)
SCr (mg/dL)	174	1.41	(0.26–8.10)
eGFR (mL/min/1.73 m^2^)	174	34.1	(4.1–173.1)
BUN (mg/dL)	174	34.0	(7.5–202.4)
CRP (mg/dL)	174	12.81	(0.04–55.73)
pH	171	7.450	(6.771–7.616)
Lactate (mmol/L)	171	3.39	(0.62–20.80)
SOFA score (at admission)	165	8	(1–18)
Site of infection			
Respiratory		35	(20.1)
Urinary tract		61	(35.1)
Abdominal		26	(14.9)
Skin and soft tissue		14	(8.0)
Blood		11	(6.3)
Others		7	(4.0)
Unknown		24	(13.8)
Duration of vasopressor use (days)	136	3	(1–27)
Duration of mechanical ventilation (days)	27	4	(2–20)
ICU length of stay (days)	174	6	(1–76)
In-hospital mortality	174	26	(14.9)

BMI, body mass index; WBC, white blood cell; RBC, red blood cell; Hb, hemoglobin; Plt, platelet; Alb, serum albumin; T-Bil, total bilirubin; SCr, serum creatinine; eGFR, estimated glomerular filtration rate; BUN, blood urea nitrogen; CRP, C-reactive protein; SOFA score, sequential organ failure assessment score; ICU, intensive care unit.

**Table 2:** Comparison of patient characteristics between the ARC and non-ARC groups (*n* = 174).

Variables		ARC (*n* = 27)		Non-ARC (*n* = 147)		*p*-Value
		*n* (%)		*n* (%)		
Patient characteristics						
Male		17	(63.0)	77	(52.4)	0.311
Age	<75 (years)	20	(74.1)	54	(36.7)	<0.001
Comorbidities						
Hypertension		7	(25.9)	70	(47.6)	0.037
Dyslipidemia		7	(25.9)	44	(30.3)	0.644
Diabetes		6	(22.2)	27	(18.6)	0.663
Laboratory data						
WBC	>8.6 (×10^3^/µL)	18	(66.7)	95	(64.6)	0.838
RBC	M < 435, F < 386 (×10^4^/µL)	20	(74.1)	93	(63.3)	0.279
Hb	M < 13, F < 12 (g/dL)	18	(66.7)	87	(59.2)	0.465
Hematocrit	M > 50, F > 44 (%)	2	(7.4)	6	(4.1)	0.360
Plt	<15.8 (×10^4^/µL)	14	(51.9)	62	(42.2)	0.352
Alb	<3.0 (g/dL)	19	(70.4)	71	(48.6)	0.038
T-Bil	>1.5 (mg/dL)	5	(18.5)	40	(27.2)	0.343
Serum sodium	<138 (mmol/L)	17	(63.0)	82	(55.8)	0.489
Serum potassium	<3.6 (mmol/L)	13	(48.1)	29	(19.7)	0.002
SCr	M > 1.063, F > 0.79 (mg/dL)	12	(44.4)	124	(84.4)	<0.001
BUN	>21 (mg/dL)	12	(44.4)	122	(83.0)	<0.001
CRP	>15.16 (mg/dL)	10	(37.0)	69	(46.9)	0.342
pH	<7.35	3	(11.5)	22	(15.2)	0.770
SOFA score	≤9	20	(80.0)	81	(57.9)	0.036

ARC, augmented renal clearance; WBC, white blood cell count; RBC, red blood cell count; Hb, hemoglobin; Plt, platelet count; Alb, serum albumin; T-Bil, total bilirubin; SCr, serum creatinine; BUN, blood urea nitrogen; CRP, C-reactive protein; SOFA score, sequential organ failure assessment score.

**Table 3:** Risk factors for ARC incidence in patients with sepsis (*n* = 174).

Variable		β	OR	95% CI			*p*-Value
Age	<75 vs. ≤75 (years)	1.558	4.748	1.683	–	13.397	0.003
Alb	<3.0 vs. ≤3.0 (g/dL)	1.301	3.672	1.282	–	10.515	0.015
Serum potassium	<3.6 vs. ≤3.6 (mmol/L)	1.648	5.196	1.908	–	14.146	0.001
SOFA score	≤9 vs. <9	1.336	3.803	1.198	–	12.067	0.023

Age, Alb, serum potassium, and SOFA score were entered into the multivariate analysis. The final multivariable model included 164 patients. β, beta coefficient; OR, odds ratio; CI, confidence interval; Alb, serum albumin; SOFA score, sequential organ failure assessment score.

## Data Availability

The original contributions presented in this study are included in the article or Supplementary Materials. Further inquiries can be directed to the corresponding author.

## Supplementary Materials

The following supporting information can be downloaded at https://www.mdpi.com/article/10.3390/jcm15145662/s1; Table S1. ROC curve analysis; Table S2. Comparison of treatments and clinical outcomes between the ARC and non-ARC groups (*n* = 174).

## Abbreviations

The following abbreviations are used in this manuscript:

Alb	albumin
ARC	augmented renal clearance
BMI	body mass index
BUN	blood urea nitrogen
CI	confidence interval
CRP	C-reactive protein
eGFR	estimated glomerular filtration rate
GFR	glomerular filtration rate
ICU	intensive care unit
MIC	minimum inhibitory concentration
RFR	renal functional reserve
SCr	serum creatinine
SOFA	sequential organ failure assessment
T > MIC	time above the minimum inhibitory concentration
WBC	white blood cell
