# Supplementary material for: Sepsis-Specific Risk Factors for Augmented Renal Clearance (ARC) and the Effect of Inflammation on Duration of ARC in Patients with Sepsis: A Single-Center Retrospective Cohort Study
Source: J Clin Med. 2026 Jul 19;15(14):5662. doi: 10.3390/jcm15145662 (PMC13413120; doi:10.3390/jcm15145662)
Supplement: Supplementary file 1 [file jcm-15-05662-s001.zip › jcm-4409069-supplementary.pdf]

Supplementary Table S1. ROC curve analysis.

**Variable/model**		**AUC**	**95% CI**			**p-value**
**Age**	**<75 (years)**	**0.687**	**0.580**	**–**	**0.793**	**0.002**
**Alb**	**<3.0 (g/dL)**	**0.609**	**0.496**	**–**	**0.721**	**0.073**
**Serum potassium**	**<3.6 (mmol/L)**	**0.642**	**0.521**	**–**	**0.764**	**0.019**
**SOFA score**	**≤9**	**0.611**	**0.498**	**–**	**0.723**	**0.078**
**Multivariable model**		**0.808**	**0.720**	**–**	**0.896**	**<0.001**

ROC curve analyses were performed separately for age, Alb, serum potassium, SOFA score and the combined multivariable model including all four variables. AUC, area under the curve; CI, confidence interval; Alb, serum albumin; SOFA score, sequential organ failure assessment score; ROC, receiver operating characteristic

Supplementary Table S2. Comparison of treatments and clinical outcomes between the ARC and non-ARC groups (*n*=174)

Variables	ARC (n=27) n (%) or median (range)		non-ARC (n=147) n (%) or median (range)		p-value
Use of vasopressor	22	(81.5)	117	(79.6)	0.822
Duration of vasopressor use (days)	3	(1–27)	3	(1–7)	0.458
Mechanical ventilation	5	(18.5)	22	(15.0)	0.639
Duration of mechanical ventilation (days)	5	(4–8)	3	(2–20)	0.606
ICU length of stay (days)	11	(2–35)	6	(1–76)	<0.001
In-hospital mortality	5	(18.5)	21	(14.3)	0.571

ARC, augmented renal clearance; ICU, intensive care unit.
